# Apparent Temperature and Cause-Specific Mortality in Copenhagen, Denmark: A Case-Crossover Analysis

**DOI:** 10.3390/ijerph8093712

**Published:** 2011-09-16

**Authors:** Janine Wichmann, Zorana Jovanovic Andersen, Matthias Ketzel, Thomas Ellermann, Steffen Loft

**Affiliations:** 1 Section of Environmental Health, Institute of Public Health, University of Copenhagen, Øster Farimagsgade 5A, Copenhagen DK-1014, Denmark; E-Mail: stl@sund.ku.dk; 2 Institute of Cancer Epidemiology, Danish Cancer Society, 49 Strandboulevarden, Copenhagen DK-2100, Denmark; E-Mail: zorana@cancer.dk; 3 Department of Environmental Sciences, Aarhus University, Frederiksborgvej 399, Roskilde DK-4000, Denmark; E-Mails: mke@dmu.dk (M.K.); tel@dmu.dk (T.E.)

**Keywords:** temperature, epidemiology, respiratory, cardiovascular, cerebrovascular, mortality, case-crossover

## Abstract

Temperature, a key climate change indicator, is expected to increase substantially in the Northern Hemisphere, with potentially grave implications for human health. This study is the first to investigate the association between the daily 3-hour maximum apparent temperature (Tapp_max_), and respiratory, cardiovascular and cerebrovascular mortality in Copenhagen (1999–2006) using a case-crossover design. Susceptibility was investigated for age, sex, socio-economic status and place of death. For an inter-quartile range (7 °C) increase in Tapp_max_, an inverse association was found with cardiovascular mortality (−7% 95% CI −13%; −1%) and none with respiratory and cerebrovascular mortality. In the cold period all associations were inverse, although insignificant.

## 1. Introduction

Anthropogenic greenhouse gas emissions are expected to raise average temperatures globally according to the fourth assessment report from the International Panel of Climate Change (IPCC), and consequently the amount of heat-related morbidity and mortality is also likely to increase in Denmark [1]. The evaluations of the relationship between key climate change factors, such as temperature, and health can help identify vulnerable populations and aid policy makers in formulating preventive actions [1].

Numerous studies reported that increased temperature increased total non-accidental mortality, both during specific heat waves [2]and over a long time period, using modern time-series or case-crossover analyses [3,4]. Few studies have investigated the relationship between temperature and cause-specific mortality [5–7] or were done in Scandinavia [8–10]. A J-shaped relationship has been found between temperature and total non-accidental and cause-specific mortality, with high temperatures (heat) having an immediate (same day or previous day) effect [3,4]. In a colder climate, the increase of global temperature may benefit health, although the wintertime increase in total non-accidental mortality may be due to infectious disease, and not a direct effect of cold weather [11]. However, few studies have investigated the relationship between decreased temperature and mortality during the colder seasons [12,13]. The overall effect of increased temperature is assumed to depend on cause of death, population characteristics, and efficiency of the health care system. Vulnerability to increased temperature may be affected by socio-economic status (SES), age, sex, level of urbanisation, household characteristics and pre-existing disease status [3,4].

Although the association between air pollution and cardiovascular disease (CVD) and respiratory disease (RD) mortality and morbidity is well established, air pollution has rarely been considered as confounder or effect modifier in studies of temperature effects [2–4]. A recent review concluded that particulate matter less than 10 μm in aerodynamic diameter (PM_10_) and ozone could be both, although the independent effect of temperature was usually withheld in analysis taken this into account [4]. Finally, few studies distinguished between in- and out-of-hospital deaths, which may also be important [14,15].

The aim of this study was to investigate the association between the daily 3-hour maximum apparent temperature (Tapp_max_) and RD, CVD and cerebrovascular disease (CBD) mortality. Susceptibility by age, sex, SES and place of death (in- or out-of-hospital) was investigated.

## 2. Methods

### 2.1. Mortality and Hospital Admission Data

Mortality and hospital admission data in the Copenhagen area (postal code < 2930, <15 km radius from the city centre, population ≈1 million) were retrieved from the Danish cause of death and hospital discharge registers, respectively. The following International Classification of Diseases 10th Revision (ICD 10) codes were included: *CVD*: angina pectoris (I20), myocardial infarction (I21–22), other acute ischemic heart diseases (I24), chronic ischemic heart disease (I25), pulmonary embolism (I26), cardiac arrest (I46), cardiac arrhythmias (I48–49), and heart failure (I50); *CBD*: intracerebral haemorrhage (I61), cerebral infarction (I62) and stroke, not specified as haemorrhage or infarction (I64); *RD*: chronic bronchitis (J41–42), emphysema (J43), chronic obstructive pulmonary disease (J44), asthma (J45) and status asthmaticus (J46).

Only primary diagnosed hospital admissions were included as outcome, but both emergency and planned hospital admissions were linked to the mortality dataset. A death was classified as in-hospital, when the hospital discharge date was the same as the date of death, and out-of-hospital when the hospital discharge date was at least one day before the date of death. Twelve deaths could not be classified due to errors in hospital discharge dates (after death).

### 2.2. Meteorological and Air Pollution Data

Meteorological and air pollution data were measured at the Copenhagen urban background monitoring station by the Department of Environmental Sciences, Aarhus University [16]. Temperature and relative humidity (RH) were measured with the HMP45a probe (Vaisala, Helsinki). Air pollution data included 24-hour averages (from midnight to midnight) of PM_10_ (Beta attenuation by SM200 monitor; Opsis, Sweden), nitrogen dioxide (NO_2_) (M 200A; API, San Diego, CA, USA) and carbon monoxide (CO) (M 300 monitor; API). NO_2_ was also reported as a daily 1-hour maximum (NO_2max_). The RH measurements have a minor error probably due to the calibration, which had a minor impact on the calculated Tapp_max_. Hence this measurement error is not likely to reduce the validity of our results (Supplementary Figures 1 and 2).

Barnett and colleagues concluded that there is no single temperature measure that is superior to others [17]. We selected Tapp_max_ as the primary exposure variable. Tapp_max_ is a construct intended to reflect the physiological experience of combined exposure to humidity and temperature and thereby better capture the response on health than temperature alone [18]. Tapp_max_ has been applied in several studies [2–4].

### 2.3. Influenza Data

Influenza epidemics data were provided by the National Serum Institute as weekly percentage of total general physician’s consultations due to influenza in Denmark, whereas city level data were not available.

### 2.4. Effect Modifier Data

Addresses of the 31,342 deceased persons were retrieved by linkage with the Danish central population registry. A recent report was published on SES groups in Copenhagen, which classified communities and the inner city neighbourhoods into four SES groups (highest, second highest, second lowest and lowest), based on household income, educational and employment status [19]. An area SES class was assigned to each person by linking the home street code to a geographical information system dataset. Nearly all (99%) of the 31,342 deceased persons lived at only one address during 1999–2006. A SES class could not be assigned to 456 people due to invalid street codes. A SES code was assigned for the valid address at which the person lived longest. In the case of more than three addresses, the mode of the area SES classes at the different addresses was assigned to that person.

### 2.5. Ethics

As this study was purely registry based, no human participants were recruited or included in experiments. Approval was granted by the proper authority, which in this case is the Danish Data Protection Agency.

### 2.6. Statistical Analysis

The time-stratified case-crossover design was applied to investigate the association between Tapp_max_ and the cause-specific mortality (in- and/or out-of-hospital) for the period 1 January 1999–1 December 2006. The case-crossover design was developed as a variant of the case-control design to study the effects of transient exposures on emergency events [20]. In this design each person’s exposure is compared in a time period just prior to a case-defining event with his/her exposure at other times [20]. Hereby, control on all measured and unmeasured personal characteristics that do not vary over a short time period is accomplished. If in addition, the control days are chosen close to the event day, personal characteristics that vary slowly over time are also controlled by matching. A time-stratified approach was applied to select the control days, defining the day of death as the case day and same day of the week in the same month and year as control days. Hence, for example, if someone died on 16 February 2000, then the control days would have been the 2, 9, 23 February 2000. With this approach even very strong confounding of exposure by seasonal patterns is controlled by design [21–24]. The association between Tapp_max_ and the cause-specific mortality were investigated using conditional logistic regression analysis (PROC PHREG in SAS 9.2, SAS Institute, Cary, NC, USA).

Models were first stratified by seasonal period (warm or cold). Public holidays were controlled for as a dichotomous variable and influenza as a continuous variable. A previous study in Copenhagen reported a linear relationship between the air pollutants and the cause-specific admissions for the period 1999–2004 [25]. The pollutants were therefore modeled as linear terms, one pollutant at a time. During 1999–2006 there were 569 and 114 days with missing values for the pollutants and meteorological variables, respectively, with a total of 625 days with missing data out of 2,922.

Individual lags of lag0 (same day exposure as day of death) to lag5 (exposure five days prior to day of death) of Tapp_max_ were investigated, as well as accumulated exposures: mean of lag0–1 (2-day simple unweighted cumulative average, CA2), and up to mean lag0–5 (CA6). Control days for lag1 to 5 were defined as for lag0. The same lag of Tapp_max_ and an air pollutant was included in a model.

There is no standard method to select a lag [26]. We selected the lag of Tapp_max_ with the lowest Akaike Information Criterion (AIC) and applied that in the stratified models. In general, the lowest AIC model had the strongest association (*i.e.*, highest absolute association measure) between Tapp_max_ and a cause-specific outcome.

A large European study observed associations between mortality and longer lags of up to CA15 for the cold period [13]. Hence, longer lags of up to CA15 for the cold period were also investigated in our study.

Hazard ratios (HR) and the 95% confidence intervals (CI) were calculated per inter-quartile range (IQR) increase in Tapp_max_ (in °C). The results are presented as the percent excess risk in cause-specific mortality per IQR increase in Tapp_max_ using the following calculation: β^(HR – 1) × 100%^, where β is the model estimate.

Due to the nature of the case-crossover design where each person is his/her own control, susceptibility cannot be investigated by including an interaction term between the susceptibility variable and Tapp_max_. Susceptibility was therefore investigated in stratified analyses by sex, age and SES groups. Age was categorised as 19–65, 66–80 and >80 years.

Sensitivity analyses were applied. The linearity and strength of the association between Tapp_max_ and a cause-specific outcome were substantiated in generalised additive Poisson time-series regression models (GAM) with the use of the *gam* procedure, *mgcv* package in R statistical software (R Development Core Team, 2010). Models were run with linear and non-linear terms for Tapp_max_, as a natural smoothing spline function with five degrees of freedom (df). Smoothing splines of calendar time (4 df/year) were used to control for long-term trend and seasonality. A spline function, defined by piecewise polynomials, has a flexible shape that is useful for adjusting for non-linear effects. The smoothness of a spline is a function of the number of degrees of freedom. We investigated whether the non-linear term for Tapp_max_ improved the models by conducting log-likelihood ratio tests. Unmeasured, unknown and potentially variable seasonal and long term patterns need to be controlled for adequately in GAM models, whilst still leaving sufficient information from which to estimate temperature effects. Other sensitivity analyses included applying the 24-hour average temperature (T_ave_) as an alternative temperature definition, whilst also adjusting for the 24-hour average RH, public holidays and influenza epidemic.

## 3. Results

The statistical analyses are based on 2,922 days with 5,973 RD, 18,816 CVD and 6,558 CBD deaths. Table 1 displays a summary of the meteorological conditions, air pollution levels and influenza epidemics during 1999–2006. None of the EU air quality limit values (PM_10_ 40 μg.m^−3^ (annual), NO_2_ 21 ppb (annual), CO 5.3 ppm (1-hour max)) were exceeded at the urban background level, but PM_10_ and NO_2_ limit values were exceeded at street level (not shown) [27].

The majority of deaths were due to CVD, followed by CBD and RD, with more deaths during the cold period (Table 1). The majority of in-hospital RD, CVD and CBD deaths occurred after emergency hospital admissions: 93%, 97% and 96%, respectively. Half of the RD and CBD deaths occurred in-hospital, whilst 66% of CVD deaths were out-of-hospital (Table 2). Regardless of place of death, the majority of RD deaths were due to chronic obstructive pulmonary disease. In-hospital CVD deaths were mostly due to acute health outcomes compared to out-of-hospital CVD deaths. Regardless of place of death, the majority of the CBD deaths were due to stroke, not specified as haemorrhage or infarction.

Supplementary Figure 3 indicates the average number of cause-specific deaths per Tapp_max_ (lag0). We did not observe a Tapp_max_ threshold in Copenhagen for which a minimum number of cause-specific deaths occurred. We therefore split a year into a warm and cold period. The warm and cold periods were defined as April–September and October–March, respectively, as for in other European cities [2,4,18,28]. Below 9 °C most days were in the cold period and at 9 °C or above most days were in the warm period (Supplementary Figure 4). So overlap of Tapp_max_ in the warm and cold periods was minimal.

CA6 of Tapp_max_ was selected as lag and applied in the stratified models (Supplementary Figure 5). In general no significant associations were observed between the air pollutants and RD, CVD or CBD mortality during 1999–2006; specifically not for the selected lag of Tapp_max_, *i.e.*, CA6. (Supplementary Figures 6 and 7). Consequently the models were not adjusted for any of the air pollutants (Tables 3 and 4). The air pollutant models were adjusted for Tapp_max_ (same lag as pollutant), public holidays and influenza.

An IQR increase in the CA6 of Tapp_max_ in the warm period was associated with an insignificant increase of 6% in RD mortality (Table 3). For an IQR increase in the CA6 of Tapp_max_ there was a significant decrease of 7% in CVD mortality during the warm period (Table 3). The association between Tapp_max_ and CVD mortality was stronger for those >80 years and those in the lowest SES group. There was no association between the CA6 of Tapp_max_ in the warm period and CBD mortality (Table 3).

In the cold period, the associations with the CA6 of Tapp_max_ were inverse, yet insignificant (Table 4). However, for RD the association reached significance for out-of-hospital deaths. For CVD and CBD, the association with Tapp_max_ reached significance in the highest and lowest SES groups, respectively.

The effect estimate of the subgroup analyses had a wide 95% CI due to the small sample sizes. The direction of the observed associations was confirmed in the GAM analyses (Table 5). The parameter estimates for the confounders (influenza, public holiday) were similar in the GAM and case-crossover analyses.

**Figure 1:**
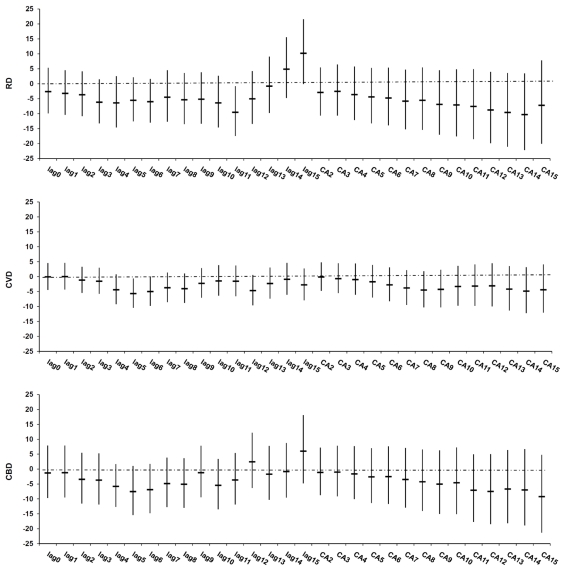
Percentage change (95% CI) in cause-specific mortality in Copenhagen per interquartile range increase in Tapp_max_ during the cold period* (1 January 1999–31 December 2006), adjusted for public holidays and influenza. * Cold period: October–March.

We did not find any evidence of a *significant* delayed effect after 6 days cumulative exposure in the cold period (Figure 1). The effect of cold on RD and CBD mortality appears to be stronger after 6 days cumulative exposure (CA6), but did not reach significance. We thus reported results for only up to CA6.

T_ave_ had similar associations with cause-specific mortality than Tapp_max_, with or without adjusting for 24-hour average RH (Supplementary Figures 8–10).

## 4. Discussion

We evaluated associations between Tapp_max_ and RD, CVD and CBD mortality in Copenhagen for the period 1999–2006. We found an apparent modest effect of increasing temperature on six subtypes of RD mortality of 0.9% per 1 °C increase in the warm period. Swedish and Norwegian studies reported stronger associations between total RD mortality (all ICD10 codes J and any place of death) and the average daily temperature (T_ave_) > 11 °C (4.3% per 1°C increase) and >10 °C (4.7% per 1 °C increase in T_ave_ over the last 7 days), respectively [8–10]. An aggregated analysis of North-Continental cities in Europe reported 6% increase in total RD mortality per 1 °C in Tapp_max_ above the city specific threshold [29,30]. Our lag structure with main apparent effects on RD mortality occurring within 5 days and increased susceptibility among the elderly corroborates the findings of North-Continental European cities [29,30].

We observed an inverse association between Tapp_max_ and the 10 subtypes of CVD mortality in the warm period: −1.0% per 1 °C increase or alternatively, 1.0% per 1 °C decrease. A Swedish study reported a weak yet positive association between total CVD mortality (all ICD10 codes I, and any place of death) and T_ave_ > 11 °C (1.1% per 1 °C increase) [10]. In the North-Continental European cities total CVD mortality increased by 2% with Tapp_max_ [29,30]. Of these cities only Dublin showed an apparently protective effect of high temperature on total mortality. Copenhagen and Dublin have quite a similar climate in the warm period [29] and it is possible that cool and rainy weather have more adverse effect on CVD mortality than the few high temperature surges.

The lack of association between Tapp_max_ and the three subtypes of CBD mortality in the warm period in Copenhagen is consistent with the findings from a large multinational European study [29]. We observed signs of protective effects of high temperature on all three causes of mortality during the cold season, consistent with other studies [5,8,12–14].

We did not observe a Tapp_max_ threshold in Copenhagen for which a minimum number of cause-specific deaths occurred (Supplementary Figure 3). However, the weight (number of days) of each Tapp_max_ is different and is taken into account in regression analyses. An insignificant linear negative and positive relationship between RD deaths, and Tapp_max_ was observed during the cold and warm periods, respectively. For CVD deaths a negative relationship was observed during both periods, although insignificant in the cold period. However, these weak associations are related to the absolute Tapp_max_, whereas our case-crossover study focuses on short-term Tapp_max_ deviations (between case and control days) within a limited period of one month.

Studies investigating susceptibility, other than age, of the temperature and cause-specific mortality relationship are scarce. We did not observed any significant adverse effect of Tapp_max_ on RD and CBD mortality by age, gender, SES or place of death in the warm period. For CVD mortality, the elderly and lowest SES group were more susceptible to increases in Tapp_max_ in the warm period. Other studies reported a stronger association between total *non-accidental mortality* and increased temperature during specific heat waves and ordinary periods among the elderly, lower SES groups and women [6,15,31]. *Total non-accidental mortality* includes deaths from a broad spectrum of causes with probable variability in sensitivity. A study that focused on extreme heat events reported a rapid increase in out-of-hospital *non-accidental* deaths, especially amongst the oldest groups [14] No extreme or long-lasting heat waves occurred in Copenhagen during 1999–2006 and this might be a possible reason why we did not observe an increase of out-of-hospital cause-specific deaths with increasing Tapp_max_.

The underlying mechanisms for increases in RD, CVD and CBD deaths after exposure to high temperatures may be due to blood flow shifts to subcutaneous areas and away from the vital organs, in an effort to cool the body [2–4]. Increased blood viscosity due to dehydration, elevated cholesterol levels and a higher sweating threshold in the elderly may trigger heat-related mortality in susceptible individuals. Factors that hamper sweating, such as high ambient humidity, reduced air currents or anticholinergic drugs reduce resistance to high temperature [2,4].

Various mechanisms are proposed to explain the increase in CVD and CBD mortality with decreasing temperatures in the cold season, such as an increase in platelet and red cell counts, blood viscosity and arterial pressure [32]. Elderly and the lowest SES groups are likely to be particularly susceptible to such cold effects compatible with our data for CBD, whereas our data on CVD showed different patterns.

Although we found no significant association between any of the air pollutants (for CA6) and cause-specific mortality, the effect estimates for lag0 of PM_10_ (per 10 μg·m^−3^ increase) and RD and CVD mortality in the cold and warm periods are similar to those of the latest meta-analysis (all year): 1.3% (95% CI 0.05%; 0.2%; pooled 18 European studies) and 0.9% (95% CI 0.05%; 1.3%; pooled 17 European studies), respectively [26]. Our 95% CIs are wider though. A significant association between PM_10_ and all cause mortality were observed for only 12 of the 33 urban centres used in the PM_10_ meta-analysis. PM_10_ was found to be an effect modifier in Australia and a confounder in Mexico and in regions throughout the United States, especially in the summer [4]. None of the other Scandinavian studies considered air pollutants as confounders or effect modifiers [8–10].

We did not find any evidence of a significant delayed effect after 6 days cumulative exposure to outdoor Tapp_max_ in the cold period, contrary to a large European study that observed associations up to CA15 [13]. Possible reasons for this inconsistency may be differences in population demographics, exposure conditions and the efficiency of the health care system.

Advantages of our study include accurate data on meteorological, air pollution and health outcome data [33,34]. Some disease misclassification is possible, but it is unlikely to be related to temperature. Another advantage is that similar results were in general observed for the association in the case-crossover and GAM analyses. The case-crossover design has some advantages over the Poisson time-series design. In Poisson time-series regression analysis, the population at risk must be very large relative to the daily number of events and the composition and size of the population at risk must not co-vary with the exposure of interest. The later assumption may not be fully met whenever the susceptible portion of the total population at risk may be increased by the cumulative effects of prior exposures or decreased by the adverse effects of prior exposures (harvesting). The case-crossover design avoids both problems as the outcome is on an individual level and not a population level (daily number of events).

One study limitation is the assumption that the outdoor temperature, humidity and air pollution measured at one site is the same across Copenhagen or even the same for each person. It is anticipated that such misclassification is more pronounced among the elderly and other frail groups who generally spend most of their time indoors. This misclassification is non-differential and should bias the effect estimates towards the null. Another limitation is that ozone data were missing for a large number of days in the period 1999–2006 and were thus not used in the analyses. Ozone is a potentially important confounder to heat effects [4].

Our results support the notion that moderate changes in ambient temperature are associated with impacts on human health even in a cool temperate climate. This association (assumed to be causal) is complex and depends on the specific health outcome (death or hospital admission), population characteristics (age, sex, SES), exposure conditions and the efficiency of the health care system, which all vary with time [1]. The results of this and many similar studies on temperature (and other key climate change factors) and health can thus not be extrapolated infinitely into the future without considering major uncertainties regarding changes in populations, the rate and intensity of projected climate change and adaptation, as stressed by the IPCC [1].

## 5. Conclusions

A moderate temperature increase had a protective effect on CVD mortality and hinted towards a slight increase in RD mortality during the warm period. In the colder months only protective effects were observed, although not statistically significant. Our results confirm that not only heat waves, but even moderate changes in ambient temperature in a Scandinavian city are associated with certain cause-specific mortality.

## Supplementary Information



## Figures and Tables

**Table 1:** Summary statistics for cause-specific mortality, air pollutant levels and meteorological conditions in Copenhagen and weekly general practice visits due to influenza in Denmark during 1 January 1999–31 December 2006.

	All year	Warm period	Cold period
Number of days	2,922	1,464	1,458
Respiratory deaths			
Mean ± SD	2 ± 2	2 ± 1	2 ± 2
Range	0–10	0–7	0–10

Cardiovascular deaths			
Mean ± SD	6 ± 3	6 ± 3	7 ± 3
Range	0–18	0–15	0–18
Cerebrovascular deaths			
Mean ± SD	2 ± 2	2 ± 2	2 ± 2
Range	0–10	0–9	0–10

Tapp_max_ (°C)			
Number of days with missing data	114	32	82
Mean ± SD	10 ± 8	16 ± 6	4 ± 5
Range	−8–30	0–30	−8–18
Percentiles			
25th	3	12	0
50th	9	16	3
75th	16	20	7
Inter-quartile range	13	8	7

PM_10_ (μg/m^3^)			
Number of days with missing data	454	266	188
Mean ± SD	27 ± 16	27 ± 14	28 ± 17
Range	0–284	1–284	0–248

NO_2_ (ppb)			
Number of days with missing data	164	109	55
Mean ± SD	12 ± 5	11 ± 4	13 ± 5
Range	2–41	3–33	2–41

NO_2max_ (ppb)			
Number of days with missing data	137	97	40
Mean ± SD	22 ± 9	21 ± 10	23 ± 9
Range	4–78	4–78	5–60

CO (ppm)			
Number of days with missing data	129	81	48
Mean ± SD	0.28 ± 0.10	0.23 ± 0.07	0.33 ± 0.10
Range	0.08–0.92	0.08–0.58	0.13–0.92

Weekly GP visits due to influenza in Denmark (%)			
Number of weeks with missing data	0	0	0
Mean ± SD	1.12 ± 1.50	0.28 ± 0.61	1.96 ± 1.65
Range	0–9.70	0–3.40	0–9.70

SD: Standard deviation; GP: General practitioner.

**Table 2:** Summary statistics for specific types of respiratory, cardiovascular and cerebrovascular deaths, by place of death during 1 January 1999–31 December 2006.

	In-hospital deaths		Out-of-hospital deaths		Total *	

	No.	%	No.	%	No.	%
**Repiratory deaths**	3,089	100.0	2,883	100.0	5,973	100.0
Simple and mucopurulent chronic bronchitis	0	0.0	7	0.2	7	0.1
Unspecified chronic bronchitis	156	5.1	313	10.9	469	7.9
Emphysema	44	1.4	73	2.5	117	2.0
Chronic obstructive pulmonary disease	2,857	92.5	2,192	76.0	5,049	84.5
Asthma	26	0.8	288	10.0	314	5.3
Status astmaticus	6	0.2	10	0.3	16	0.3
**Cardiovascular deaths**	6,310	100.0	12,502	100.0	18,816	100.0
Angina pectoris	26	0.4	61	0.5	87	0.5
Acute myocardial infarction	2,517	39.9	3,064	24.5	5,581	29.7
Subsequent myocardial infarction	154	2.4	165	1.3	319	1.7
Other acute ischemic heart diseases	8	0.1	8	0.1	16	0.1
Chronic ischemic heart disease	1,387	22.0	5,244	41.9	6,631	35.2
Pulmonary embolism	337	5.3	304	2.4	641	3.4
Cardiac arrest	143	2.3	1,013	8.1	1,156	6.1
Atrial fibrillation and flutter	544	8.6	496	4.0	1,040	5.5
Other cardiac arrhythmias	27	0.4	92	0.7	119	0.6
Heart failure	1,167	18.5	2,055	16.4	3,222	17.1
**Cerebrovascular deaths**	3,469	100.0	3,082	100.0	6,558	100.0
Intracerebral haemorrhage	1,128	32.5	389	12.6	1,517	23.1
Cerebral infarction	595	17.2	345	11.2	940	14.3
Stroke, not specified as haemorrhage or infarction	1,746	50.3	2,348	76.2	4,094	62.4

**Total**	**12,868**		**18,467**		**31,347**	

* One, four and seven RD, CVD and CBD deaths could not be classified as in- or out-of-hospital deaths, due to errors in hospital discharge dates (after death).

**Table 3:** Association between Tapp_max_ (in °C) and mortality, by cause of death, expressed as percentage increase in risk (%) and 95% confidence intervals per inter-quartile increase in the 6-day cumulative average of Tapp_max_ (in °C) during warm period of 1 January 1999–31 December 2006 in Copenhagen.

	Respiratory disease					Cardiovascular disease					Cerebrovascular disease				
	
	IQR	n	%	95% CI		IQR	n	%	95% CI		IQR	n	%	95% CI	
**All**	7	2,431	6.3	−5.4	19.4	7	7,976	−**6.9**	−**12.7**	−**0.6**	7	2,834	2.0	−8.6	13.8
**Age categories**															
≤ 65 years	8	234	−3.7	−36.5	46.1	7	871	7.5	−11.3	30.4	7	249	21.3	−16.7	76.7
66–80 years	7	1,145	5.3	−11.1	24.8	7	2,225	−9.0	−19.5	2.9	7	788	7.1	−12.8	31.6
> 80 years	8	1,052	12.3	−8.4	37.6	7	4,880	−**8.3**	−**15.7**	−**0.4**	7	1,797	−2.6	−15.2	11.9
**Sex**															
Women	7	1,422	1.0	−13.3	17.7	7	4,286	−4.9	−13.0	3.9	7	1,780	1.0	−12.2	16.2
Men	8	1,009	16.3	−5.2	42.8	7	3,690	−9.1	−17.4	0.0	7	1,054	3.9	−13.0	24.0
**Socio-economic status**															
Lowest	7	882	4.8	−13.7	27.4	7	2,667	−**11.2**	−**20.7**	−**0.6**	7	876	13.6	−6.8	38.3
Second lowest	8	677	−12.5	−31.9	12.4	7	2,000	−6.7	−17.9	6.0	7	725	−5.4	−23.7	17.4
Second highest	8	572	30.2	−0.7	70.7	8	2,166	−1.5	−14.7	13.6	7	801	−10.5	−27.3	10.2
Highest	8	265	20.8	−19.6	81.5	7	1,034	−7.0	−22.5	11.6	8	396	16.0	−17.5	63.1
**Place of death**															
In-hospital	8	1,242	1.1	−16.0	21.7	7	2,629	−9.4	−19.1	1.4	7	1,488	−7.0	−20.2	8.4
Out-of-hospital	7	1,188	12.1	−5.1	32.5	7	5,345	−5.6	−12.8	2.2	7	1,342	11.5	−4.8	30.7

* Adjusted for day of the week, public holidays and influenza rates.

**Table 4:** Association between Tapp_max_ (in °C) and mortality, by cause of death, expressed as percentage increase in risk (%) and 95% confidence intervals per inter-quartile increase in the 6-day cumulative average of Tapp_max_ (in °C) during cold period of 1 January 1999–31 December 2006 in Copenhagen.

	Respiratory disease					Cardiovascular disease					Cerebrovascular disease				
	
	IQR	n	%	95% CI		IQR	n	%	95% CI		IQR	n	%	95% CI	
**All**	6	2,854	−4.8	−13.9	5.4	6	8,777	−2.7	−8.2	3.1	6	3,010	−2.5	−11.7	7.6
**Age categories**															
≤ 65 years	6	318	4.7	−22.7	41.8	6	927	−13.7	−28.0	3.5	7	221	26.1	−15.5	88.1
66–80 years	6	1,349	1.9	−12.1	18.2	6	2,415	5.9	−5.2	18.2	6	885	−1.6	−18.1	18.1
> 80 years	6	1,187	−13.4	−25.9	1.2	6	5,435	−4.5	−11.3	2.8	6	1,904	−5.7	−16.8	6.8
**Sex**															
Women	6	1,720	−1.7	−13.6	12.0	6	4,769	−0.3	−7.8	7.9	6	1,970	−0.3	−11.9	12.8
Men	6	1,134	−9.3	−22.8	6.5	6	4,008	−5.6	−13.3	2.9	6	1,040	−6.0	−20.3	10.8
**Socio-economic status**															
Lowest	6	1,034	−6.2	−20.2	10.4	6	2,996	−2.1	−11.3	8.1	6	937	−**19.2**	−**32.4**	−**3.5**
Second lowest	6	744	−1.2	−19.7	21.5	6	2,121	0.5	−10.7	13.0	6	807	11.8	−7.6	35.3
Second highest	6	701	−10.8	−27.2	9.4	6	2,362	0.3	−10.3	12.1	6	806	−2.8	−19.9	17.8
Highest	6	341	4.5	−22.1	40.2	6	1,144	−**16.2**	−**28.5**	−**1.7**	6	419	11.1	−14.3	44.2
**Place of death**															
In-hospital	6	1,494	5.0	−8.7	20.7	6	2,978	−6.8	−15.6	2.8	6	1,603	−4.5	−16.6	9.2
Out-of-hospital	6	1,360	−**14.4**	−**26.0**	−**0.9**	6	5,797	−0.6	−7.4	6.8	6	1,406	0.0	−13.5	15.7

* Adjusted for day of the week, public holidays and influenza rates.

**Table 5:** Association between Tapp_max_ (in °C) and mortality, by cause of death, expressed as percentage increase in risk (%) and 95% confidence intervals per inter-quartile increase in the 6-day cumulative average of Tapp_max_ (in °C) during 1 January 1999 – 31 December 2006 in Copenhagen.: Generalised additive Poisson time-series regression models.

	Respiratory disease					Cardiovascular disease					Cerebrovascular disease				
	
	IQR	n*	%	95% CI		IQR	n	%	95% CI		IQR	n	%	95% CI	
**Warm**	7	1,342	3.3	−5.4	12.8	7	1,342	−4.4	−8.9	0.4	7	1,342	−1.3	−9.1	7.2
**Cold**	6	1,271	−**8.0**	−**14.1**	−**1.5**	6	1,271	−**7.5**	−**10.9**	−**4.0**	6	1,271	−0.9	−7.3	6.0

Models adjusted for calendar time (4 df/year), day of the week, public holidays and influenza rates.

* Number of days in GAM. Fewer days than in Table 1 due to missing data for 6-day cumulative average of Tapp_max_.

## References

[1] Parry ML, Canziani OF, Palutikof JP, van der Linden PJ, Hanson CE (2007). Contribution of Working Group II to the Fourth Assessment Report of the Intergovernmental Panel on Climate Change, 2007.

[2] Kovats RS, Hajat S (2008). Heat stress and public health: A critical review. Ann Rev Public Health.

[3] Basu R, Samet JM (2002). Relation between elevated ambient temperature and mortality: A review of the epidemiologic evidence. Epidemiol Rev.

[4] Basu R (2009). High ambient temperature and mortality: A review of epidemiologic studies from 2001 to 2008. Environ Health.

[5] Braga A, Zanobetti A, Schwartz J (2002). The effect of weather on respiratory and cardiovascular deaths in 12 US cities. Environ Health Perspect.

[6] Medina-Ramon M, Zanobetti A, Cavanagh DP, Schwartz J (2006). Extreme temperatures and mortality: Assessing effect modification by personal characteristics and specific cause of death in a multi-city case-only analysis. Environ Health Perspect.

[7] Barnett AG (2007). Temperature and cardiovascular deaths in the US elderly: Changes over time. Epidemiology.

[8] Nafstad P, Skrondal A, Bjertness E (2001). Mortality and temperature in Oslo, Norway, 1990–1995. Euro J Epidemiol.

[9] Näyhä S (2007). Heat mortality in Finland in the 2000s. Int J Circum Health.

[10] Rocklöv J, Forsberg B (2008). The effect of temperature on mortality in Stockholm 1998–2003: A study of lag structures and heatwave effects. Scand J Public Health.

[11] Reichert TA, Simonsen L, Sharma A, Pardo SA, Fedson DS, Miller MA (2004). Influenza and the winter increase in mortality in the United States, 1959–1999. Am J Epidemiol.

[12] Eurowinter Group (1997). Cold exposure and winter mortality from ischaemic heart disease, cerebrovascular disease, respiratory disease, and all causes in warm and cold regions of Europe. Lancet.

[13] Analitis A, Katsouyanni K, Biggeri A, Baccini M, Forsberg B, Bisanti L, Kirchmayer U, Ballester F, Cadum E, Goodman PG (2008). Effects of cold weather on mortality: Results from 15 European cities within the PHEWE project. Am J Epidemiol.

[14] O’Neill MS, Zanobetti A, Schwartz J (2003). Modifiers of the temperature and mortality association in seven US cities. Am J Epidemiol.

[15] Stafoggia M, Forastiere F, Agostini D, Biggeri A, Bisanti L, Cadum E, Caranci N, de’Donato F, De Lisio S, De Maria M (2006). Vulnerability to heat-related mortality: A multicity, population-based, case-crossover analysis. Epidemiology.

[16] Ellermann T, Nordstr⊘m C, Brandt J, Christensen J, Ketzel M, Jensen SS (2011). The Danish Air Quality Monitoring Programme. Annual Summary for 2010, Technical Report No 836.

[17] Barnett AG, Tong S, Clements ACA (2010). What measure of temperature is the best predictor of mortality?. Environ Res.

[18] Michelozzi P, Accetta G, De Sario M, D’Ippoliti D, Marino C, Baccini M, Biggeri A, Anderson HR, Katsouyanni K, Ballester F (2009). High temperature and hospitalizations for cardiovascular and respiratory causes in 12 European cities. Am J Respir Crit Care Med.

[19] Danish Health Review for Regions and Communes (2008). Sundhedsprofil for Region og Kommuner. 2008.

[20] Maclure M (1991). The case-crossover design: A method for studying transient effects on the risk of acute events. Am J Epidemiol.

[21] Bateson TF, Schwartz J (1991). Control for seasonal variation and time trend in case-crossover studies of acute effects of environmental exposures. Epidemiology.

[22] Lee JT, Kim H, Schwartz J (2002). Bidirectional case-crossover studies of air pollution: Bias from skewed and incomplete waves. Environ Health Perspect.

[23] Bateson TF, Schwartz J (2001). Selection bias and confounding in case-crossover analyses of environmental time-series data. Epidemiology.

[24] Levy D, Lumley T, Sheppard L, Kaufman J, Checkoway H (2001). Referent selection in case crossover analyses of acute health effects of air pollution. Epidemiology.

[25] Andersen ZJ, Wahlin P, Raaschou-Nielsen O, Scheike T, Loft S (2007). Ambient particle source apportionment and daily hospital admissions among children and elderly in Copenhagen. J Expo Sci Environ Epidemiol.

[26] Anderson HR, Atkinson RW, Peacock JL, Marston L, Konstantinou K (2004). Meta-Analysis of Time Series Studies and Panel Studies of Particulate Matter (PM) and Ozone (O_3_). 2004.

[27] European Commission Environment DG (2008). Directive on Ambient Air Quality and Cleaner Air for Europe (Directive 2008/50/EC).

[28] Mercer JB (2003). Cold—An underrated risk factor for health. Environ Res.

[29] Baccini M, Biggeri A, Accetta G, Kosatsky T, Katsouyanni K, Analitis A, Anderson HR, Bisanti L, D’Ippoliti D, Danova J (2008). Heat effects on mortality in 15 European cities. Epidemiology.

[30] Baccini M, Tom K, Biggeri A (2011). Impact of heat on mortality in 15 European cities: Attributable deaths under different weather scenarios. J Epidemiol Community Health.

[31] O’Neill MS, Ebi KL (2009). Temperature extremes and health: Impacts of climate variability and change in the United States. J Occup Environ Med.

[32] McArthur K, Dawson J, Walters M (2010). What is it with weather and stroke?. Expert Rev Neurotherap.

[33] Mathers CD, Fat DM, Inoue M, Rao C, Lopez AD (2005). Counting the dead and what they died from: An assessment of the global status of cause of death data. Bull World Health Organ.

[34] Helweg-Larsen K (2011). The Danish register of causes of death. Scand J Public Health.

