# Supplementary material for: Apparent Temperature and Cause-Specific Mortality in Copenhagen, Denmark: A Case-Crossover Analysis
Source: Int J Environ Res Public Health. 2011 Sep 16;8(9):3712–27. doi: 10.3390/ijerph8093712 (PMC3194112; doi:10.3390/ijerph8093712)
Supplement: Supplementary file 1 [file ijerph-08-03712-s001.pdf]

Supplementary Results

1. Measurement Error in 24-hour Relative Humidity Measurements

The 24-hour relative humidity measurements have a minor error, probably due to the calibration (Supplementary Figure 1).

The 24-hour relative humidity values decreased by about 10% during January 1999 to December 2006 and had a minor impact on the calculated apparent temperature (Supplementary Figure 2). Hence, the error in the 24-hour relative humidity measurements is not likely to reduce the validity of the results.

Apparent temperature is a construct intended to reflect the physiological experience of combined exposure to humidity and temperature and thereby better capture the response on health than temperature alone [1].

Saturation vapour pressure

$$= 6.112 \times 10^{(7.5 \times \text{temperature °C}/(237.7 + \text{temperature °C})} \qquad \text{Equation (1)}$$

Actual vapour pressure

$$= (\text{relative humidity (\%)} \times \text{saturation vapour pressure})/100 \qquad \text{Equation (2)}$$

Dew point temperature °C

$$= (-430.22 + 237.7 \times \ln(\text{actual vapour pressure}))/(-\ln(\text{actual vapour pressure}) + 19.08) \qquad \text{Equation (3)}$$

Apparent temperature °C

$$= -2.653 + (0.994 \times \text{temperature °C}) + 0.0153 \times (\text{dew point temperature °C}) \qquad \text{Equation (4)}$$

2. $Tapp_{max}$ Threshold

Supplementary Figure 3 indicates the daily average number of cause-specific deaths per $Tapp_{max}$ (lag0). We did not observe a $Tapp_{max}$ threshold in Copenhagen for which a minimum number of cause-specific deaths occurred. We therefore split a year into a warm (April–September) and cold period (October–March), as done for Stockholm and other European cities [1-3].

Below 9 °C most days were in the cold period and at 9 °C or above most days were in the warm period (Supplementary Figure 4). So overlap of $Tapp_{max}$ in the warm and cold periods was minimal.

3. Lag Selection: $Tapp_{max}$

Supplementary Figure 5 illustrates the % change in the cause-specific mortality per IQR increase in the different lags of $Tapp_{max}$ during the warm and cold periods, respectively, after adjusting for public holidays and weekly influenza rates, but not for any of the pollutants.

In general the strongest statistically significant association and model with the lowest Akaike Information Criterion were observed between the 6-day cumulative average (CA6) of $Tapp_{max}$ and the cause-specific mortality. CA6 of $Tapp_{max}$ was selected as lag and applied that in the stratified models.

Supplementary Figure 1. 24-hour average relative humidity measurements (lag0) in Copenhagen during 1 January 1999–31 December 2006.

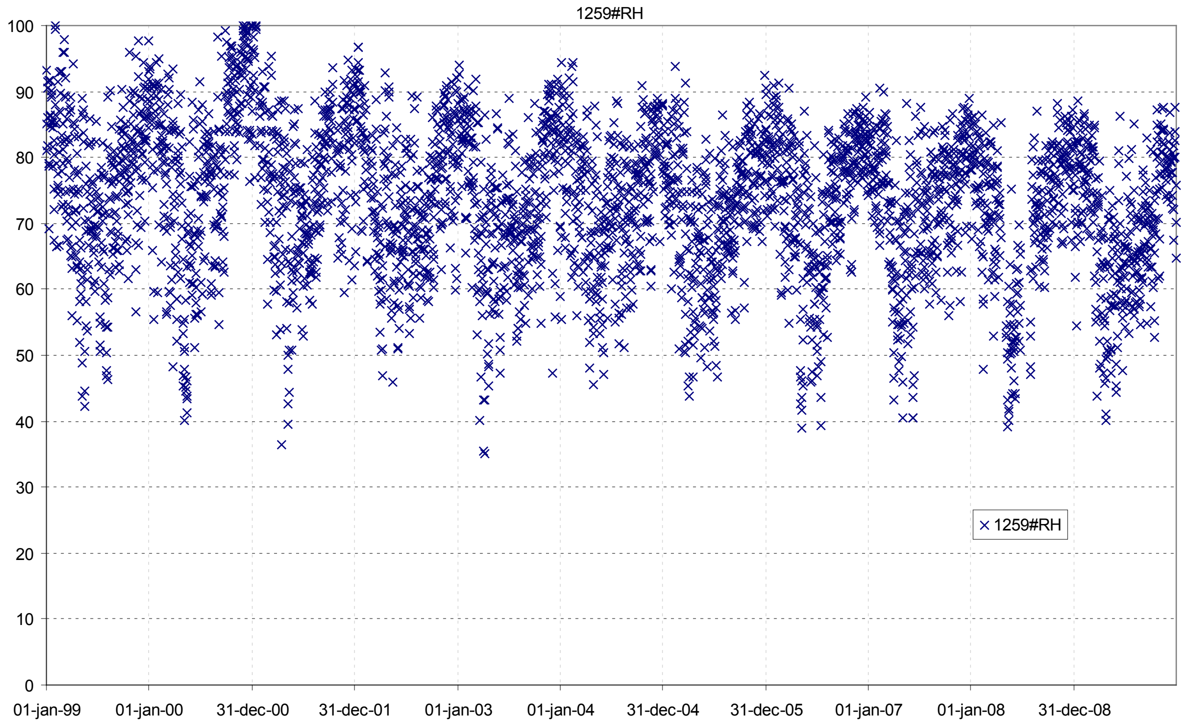

Supplementary Figure 2. Theoretical relationship between apparent temperature, temperature and relative humidity.

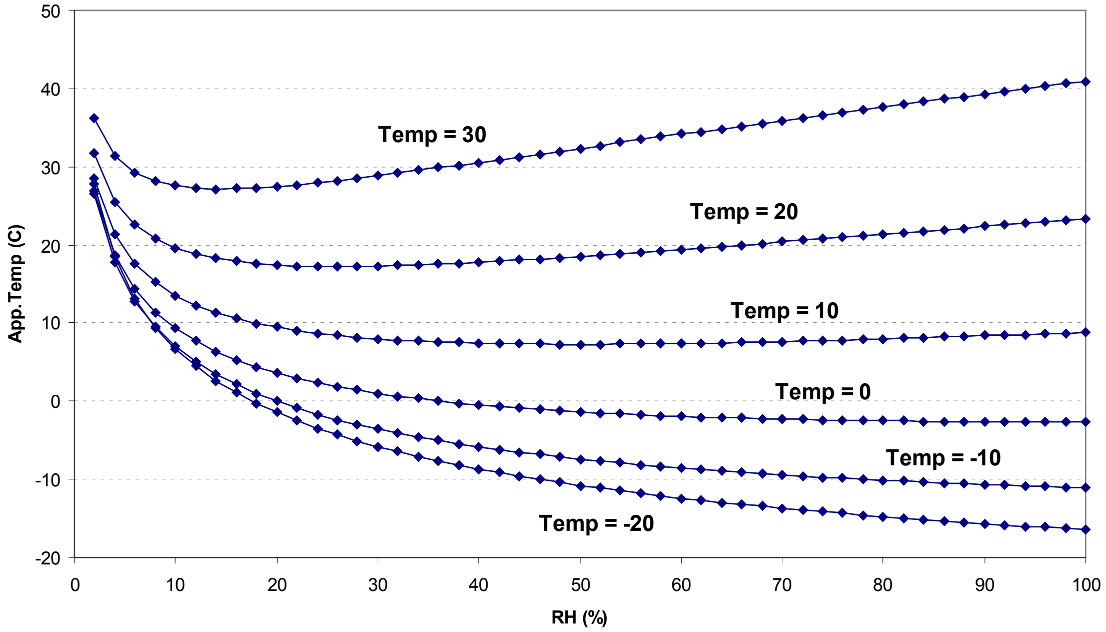

Supplementary Figure 3. Total and daily average number of cause-specific deaths per same day Tapp$_{max}$ (lag0) in Copenhagen during 1 January 1999–31 December 2006.

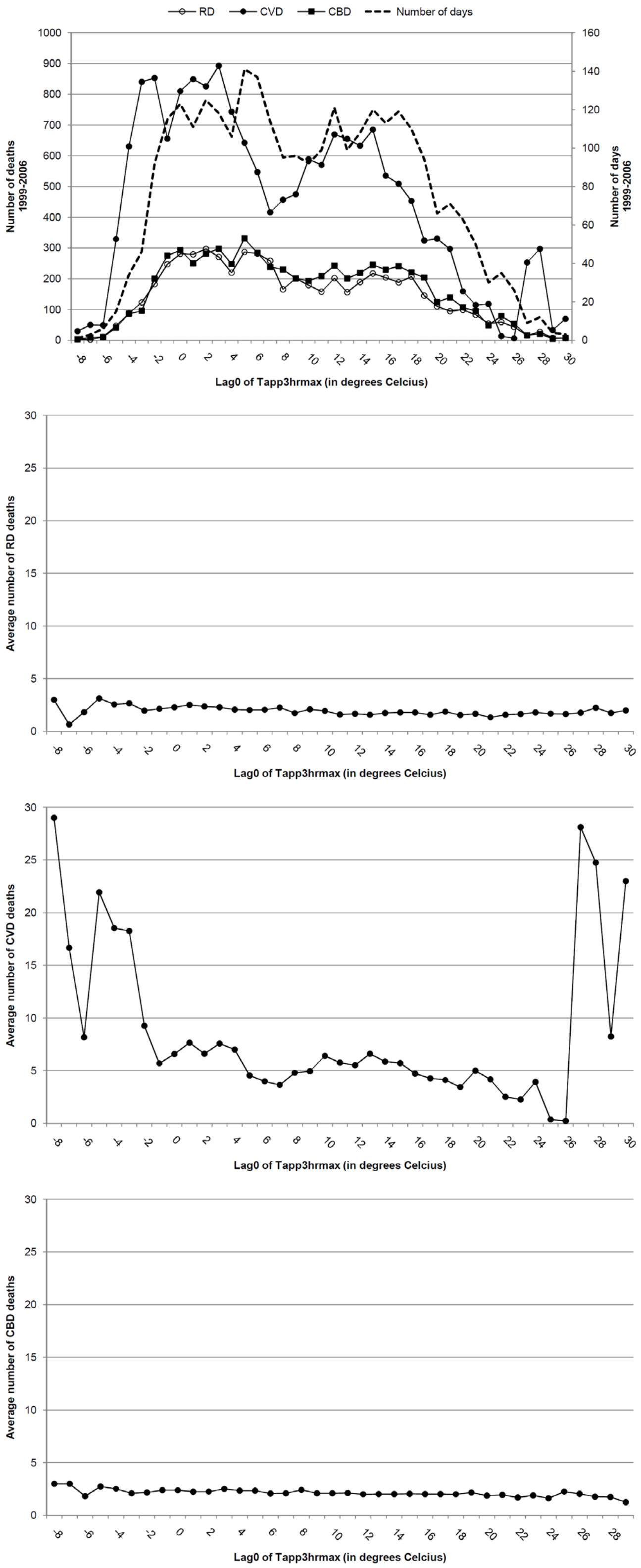

Supplementary Figure 4. Number of days with a specific $Tapp_{max}$ (lag0) in Copenhagen during the warm and cold period * (1 January 1999–31 December 2006).

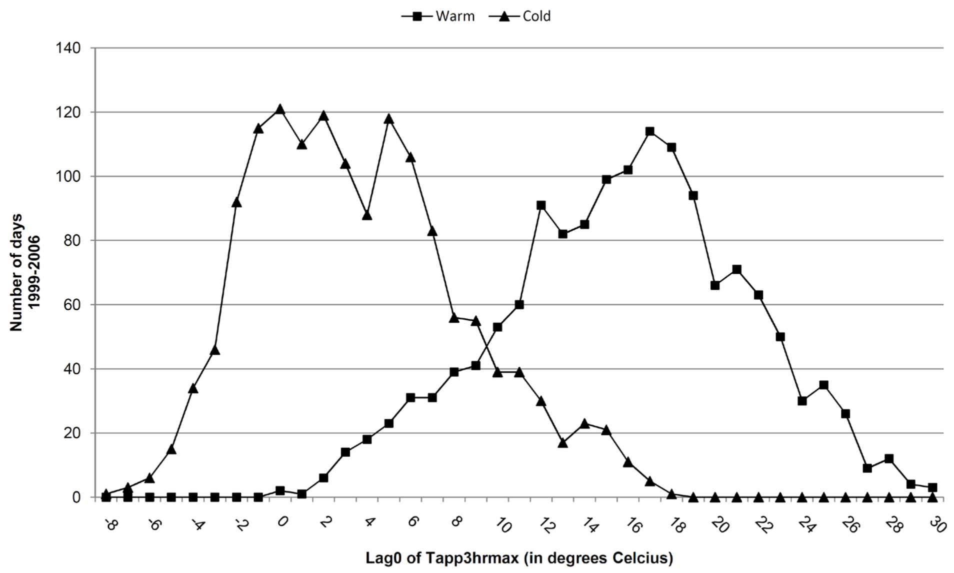

* Warm period: April–September, Cold period: October–March.

Supplementary Figure 5. Percentage change (95% CI) in cause-specific mortality in Copenhagen per inter-quartile range increase in $Tapp_{max}$ during the warm and cold period* (1 January 1999–31 December 2006), adjusted for public holidays and influenza

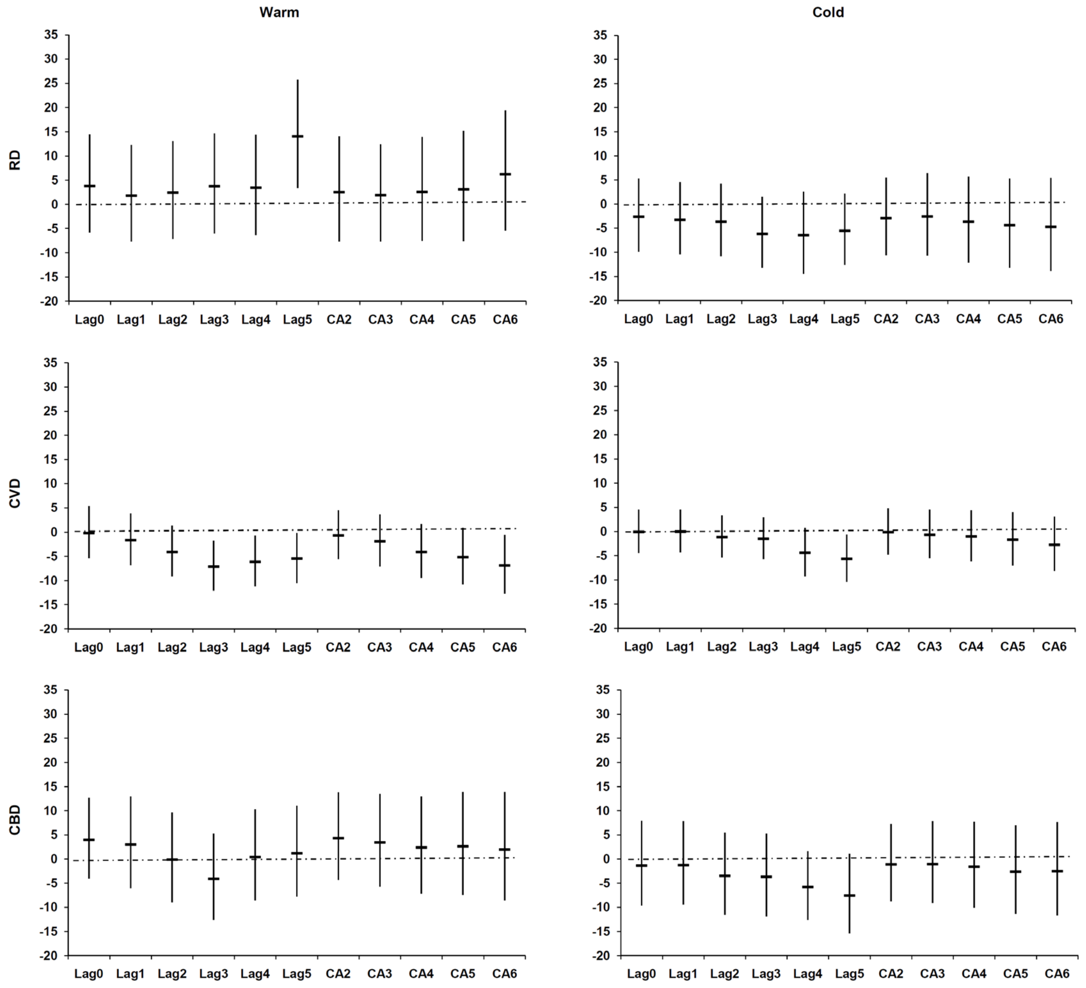

* Warm period: April–September, Cold period: October–March.

Supplementary Figure 6. Percentage change (95% CI) in cause-specific mortality in Copenhagen per inter-quartile range increase in PM_{10}, NO_2, NO_{2max} and CO during the warm period (1 January 1999–31 December 2006), adjusted for $Tapp_{max}$, public holidays and influenza*.

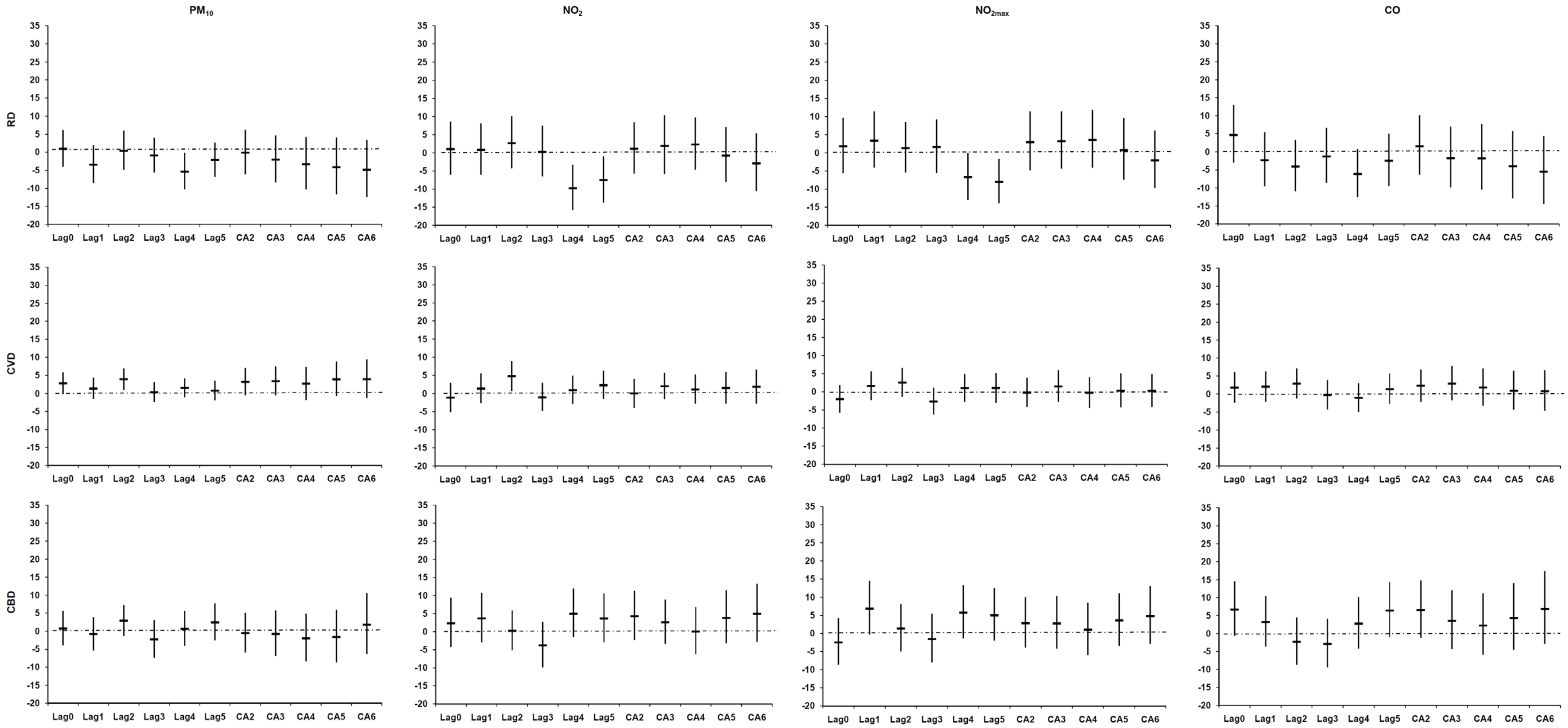

* Same lag as pollutant, Warm period: April–September.

Supplementary Figure 7. Percentage change (95% CI) in cause-specific mortality in Copenhagen per inter-quartile range increase in PM_{10}, NO_2, NO_{2max} and CO during the cold period (1 January 1999–31 December 2006), adjusted for $Tapp_{max}$, public holidays and influenza*.

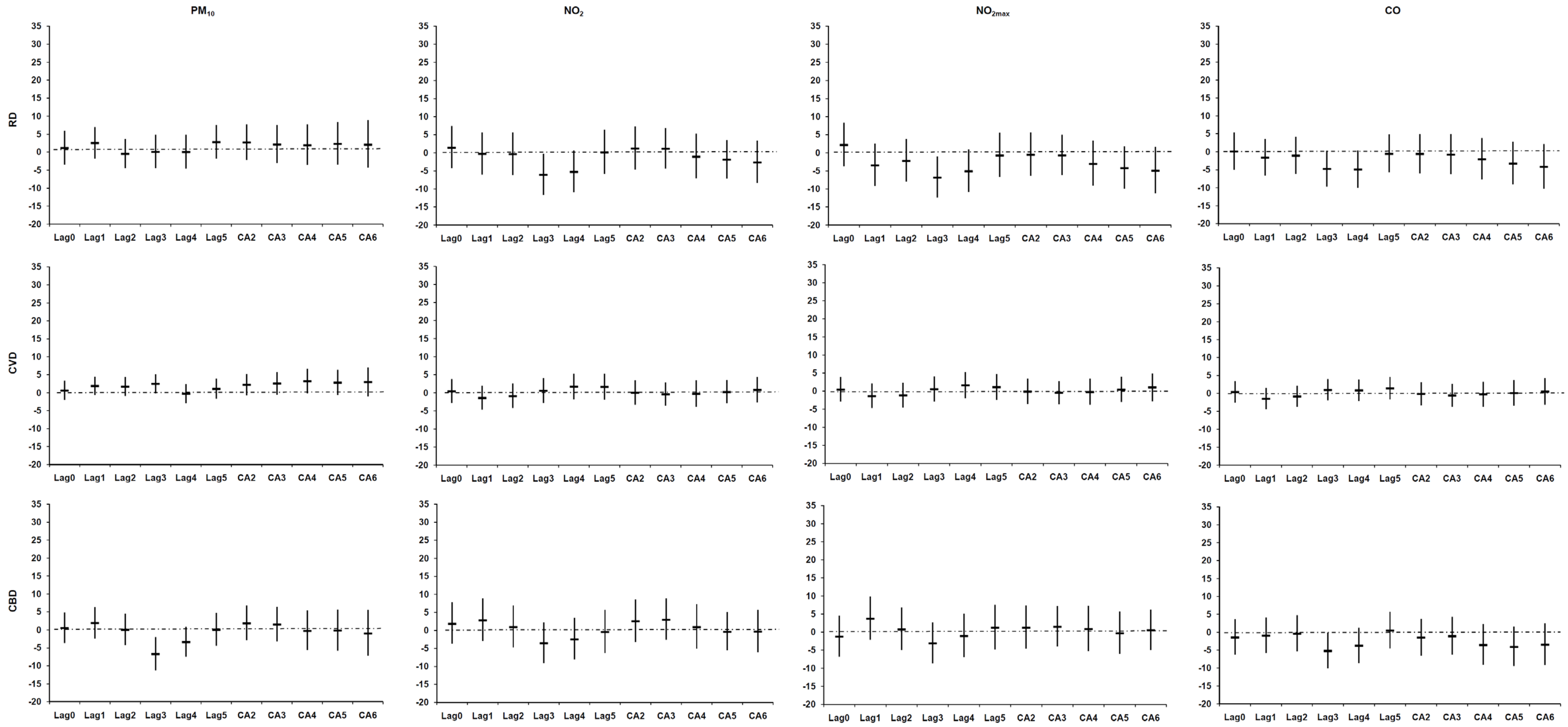

* Same lag as pollutant, Cold period: October–March.

5. Sensitivity Analyses: Alternative Temperature Definition

Barnett and colleagues concluded that there is no single temperature indicator that is superior to others [4]. We also observed this in our results.

The robustness of the observed $Tapp_{max}$ associations (Tables 3 and 4, Supplementary Figure 5) was confirmed in models with the alternative temperature definition: 24-hour average temperature (T_{ave}). Supplementary Figure 8 illustrates the % change in the cause-specific mortality per IQR increase in the different lags of T_{ave} during the warm and cold periods, respectively, after adjusting for 24-hour mean relative humidity (RH_{ave}), public holidays and weekly influenza rates, but not for any of the pollutants. A similar lag structure was observed as in the $Tapp_{max}$ models (Supplementary Figure 5).

Supplementary Figure 8. Percentage change (95% CI) in cause-specific mortality in Copenhagen per inter-quartile range increase in the 24-hour average temperature during the warm and cold period (1 January 1999–31 December 2006), adjusted for public holidays, influenza and 24-hour average relative humidity*.

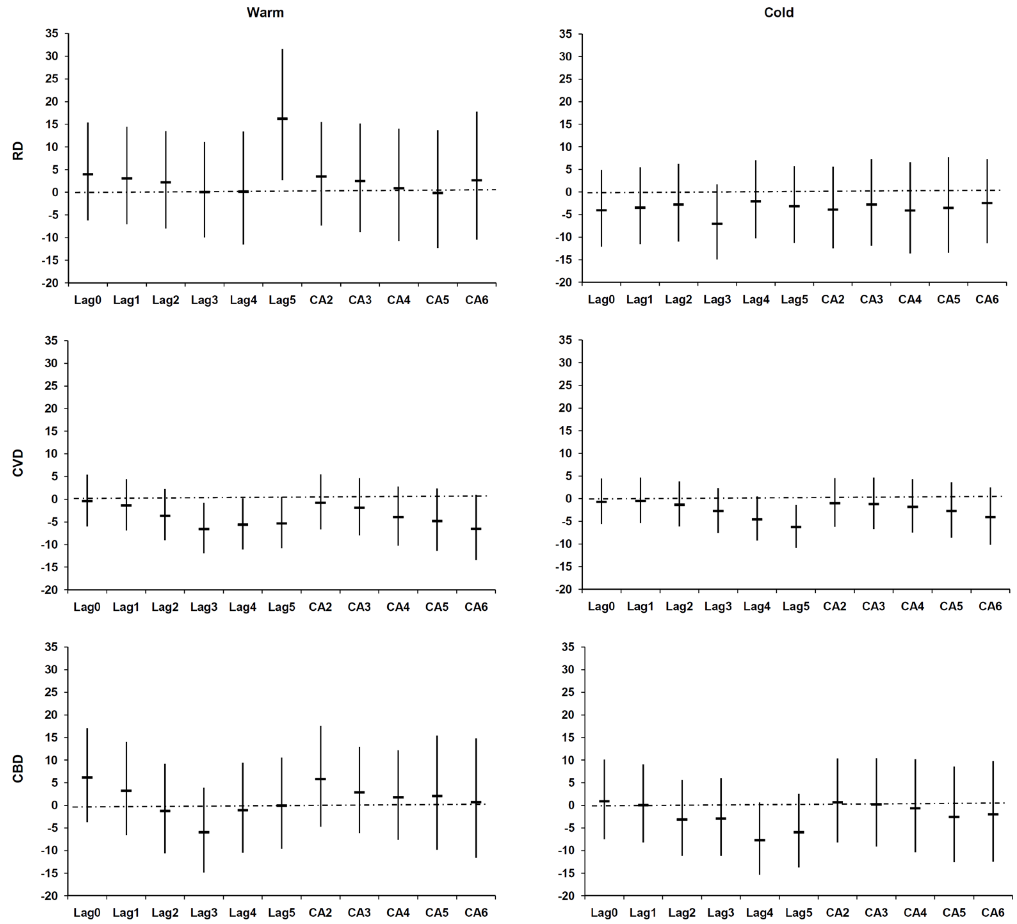

* Same lag as 24-hour average temperature, Warm period: April–September, Cold period: October–March.

Supplementary Figure 9 illustrates the % change in the cause-specific mortality per IQR increase in the different lags of RH_{ave} during the warm and cold periods, respectively, after adjusting for T_{ave}, public holidays and weekly influenza rates, but not for any of the pollutants. RH_{ave} was only significantly associated with RD mortality in the warm period. A large multicity study from the United States also found that humidity had a weak and inconsistent association with RD and CVD mortality [5].

Supplementary Figure 9. Percentage change (95% CI) in cause-specific mortality in Copenhagen per inter-quartile range increase in the 24-hour average relative humidity during the warm and cold period (1 January 1999–31 December 2006), adjusted for public holidays, influenza and 24-hour average temperature*.

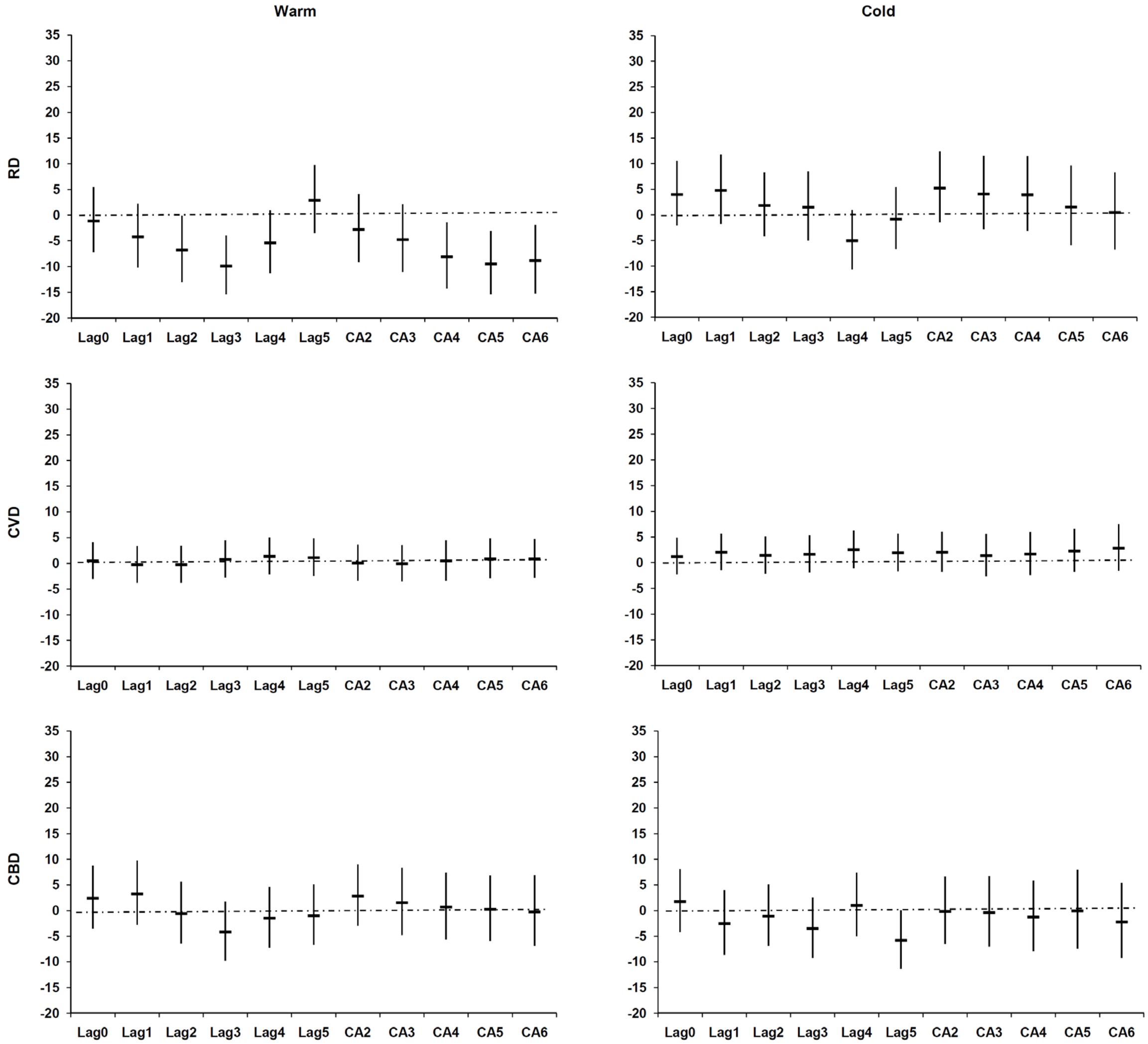

* Same lag as 24-hour average relative humidity, Warm period: April–September, Cold period: October–March.

Supplementary Figure 10 illustrates the % change in the cause-specific mortality per IQR increase in the different lags of T_{ave} during the warm and cold periods, respectively, after adjusting for public holidays and weekly influenza rates, but not for RH_{ave} nor for any of the pollutants. A similar lag structure was observed as in the $Tapp_{max}$ models (Supplementary Figure 5).

Supplementary Figure 10. Percentage change (95% CI) in cause-specific mortality in Copenhagen per inter-quartile range increase in the 24-hour average temperature during the warm and cold period (1 January 1999–31 December 2006), adjusted for public holidays and influenza, but not for 24-hour average relative humidity*.

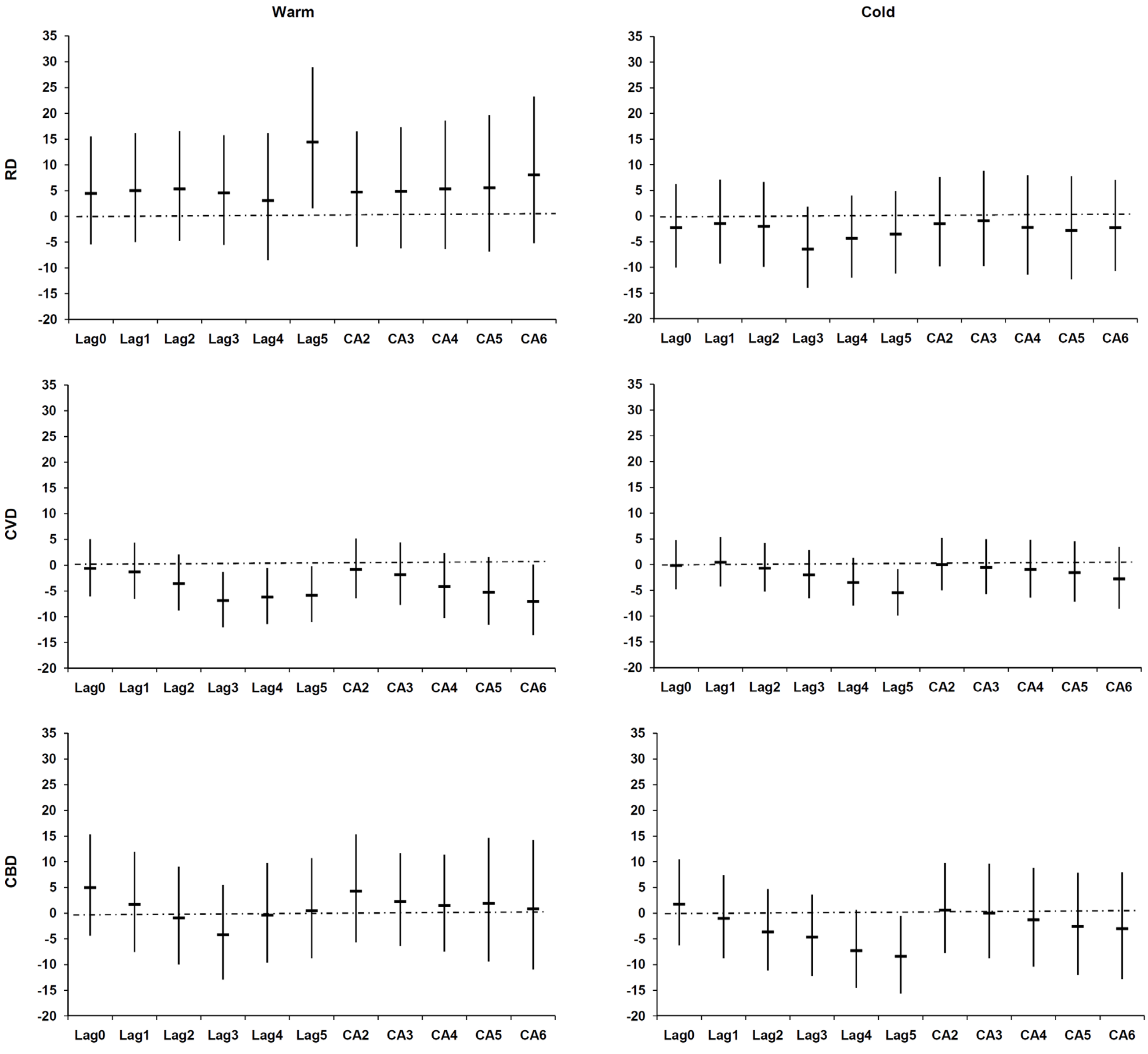

* Warm period: April–September, Cold period: October–March.

Supplementary References

1. Michelozzi, P.; Accetta, G.; De Sario, M.; D'Ippoliti, D.; Marino, C.; Baccini, M.; Biggeri, A.; Anderson, H.R.; Katsouyanni, K.; Ballester, F.; *et al.* High temperature and hospitalizations for cardiovascular and respiratory causes in 12 European cities. *Am. J. Respir. Crit. Care Med.* **2009**, *179*, 383-389.
2. Basu. R.; Samet, J.M. Relation between elevated ambient temperature and mortality: A review of the epidemiologic evidence. *Epidemiol. Rev.* **2002**, *24*, 190-202.
3. Basu, R. High ambient temperature and mortality: A review of epidemiologic studies from 2001 to 2008. *Environ. Health* **2009**, *8*, doi:10.1186/1476-069X-8-40.
4. Barnett, A.G.; Tong, S.; Clements, A.C.A. What measure of temperature is the best predictor of mortality? *Environ. Res.* **2010**, *110*, 604-611.
5. Braga, A.; Zanobetti, A.; Schwartz, J. The effect of weather on respiratory and cardiovascular deaths in 12 US cities. *Environmental Health Perspectives* **2002**, *110*, 859-863.
